# Resveratrol mitigates lipopolysaccharide-mediated acute inflammation in rats by inhibiting the TLR4/NF-κBp65/MAPKs signaling cascade

**DOI:** 10.1038/srep45006

**Published:** 2017-03-21

**Authors:** Guangxi Wang, Zhiqiang Hu, Qiuting Fu, Xu Song, Qiankun Cui, Renyong Jia, Yuanfeng Zou, Changliang He, Lixia Li, Zhongqiong Yin

**Affiliations:** 1 Natural Medicine Research Center, College of Veterinary Medicine, Sichuan Agricultural University, Chengdu, 611130, China; 2 Key laboratory of Animal Disease and Human Health of Sichuan Province, Sichuan Agricultural University, Chengdu, 611130, China

## Abstract

Resveratrol (RSV) is a natural compound exhibiting anti-inflammatory effect, but the anti-inflammatory mechanism has not been fully understood. This study is aimed to evaluate the anti-inflammatory activity and mechanism of RSV in lipopolysaccharides-induced rats’ model. The visceral wet/dry weight ratios and the changes of hematologic and biochemical indices indicated that LPS- stimulation mainly caused damages to liver and lung, while pretreatment with RSV could alleviate the lesions. The cytokine assays showed that RSV could markedly decrease the production of proinflammatory mediators and cytokines (IL-1, IL-1β, IL-6, NO, iNOS and COX-2), and increase the expression of anti-inflammatory mediator (IL-10). RSV could inhibit TLR4 signaling pathway by down-regulating the mRNA levels of MyD88 and TRAF6, and suppressing the TLR4 protein. RSV could inhibit the signaling cascades of NF-κBp65 and MAPKs through down-regulating the mRNA levels of IκBα, p38MAPK, JNK, ERK1, ERK2 and ERK5 in liver and lung, and suppressing the dynamic changes of proteins and phosphorylated proteins including IκBα, NF-κBp65, p38MAPK, JNK, ERK1/2 and ERK5 from tissue’s cytoplasm to nucleus. In conclusion, RSV possessed a therapeutic effect on LPS-induced inflammation in rats and the mechanism mainly attributed to suppressing the signaling cascades of NF-κBp65 and MAPKs by inhibiting the TLR4 signaling pathway.

Inflammation is a self-protection of microcirculation against harmful stimuli, including physical, chemical and biological stimuli^1^. However, excessive and uncontrolled inflammation may cause severe tissue damages and secondary inflammatory injuries, even producing damages to DNA^2^,^3^. Therefore, anti- inflammatory treatments are urgently needed. Nonsteroidal anti-inflammatory drugs (NSAIDs) are the main methods for treating inflammatory diseases, but long-term use often causes serious side effects that limit their applications^4^,^5^.

Lipopolysaccharides (LPS) is the main glycolipid component of endotoxin which derives from Gram-negative bacteria cell walls that can cause damages to the host, including generation of inflammation, deleterious effects on organs, septic shock and death^6^. Previous researches have confirmed that LPS induces inflammatory response mainly by activating Toll-like receptor 4 (TLR4) signaling pathway. TLR4 belongs to a large family of evolutionary conserved transmembrane proteins implicated in kinds of immune response^7^ and can be activated by agonists and bacterial lipopeptides. Myeloid differentiation primary response gene 88 (MyD88) is one of the main adaptor proteins for TLR4 promoting signal transduction^8^. The MyD88-dependent pathway activates the mitogen-activated protein kinase pathways (MAPKs)^9^,^10^ via recruiting interleukin-1 receptor associated kinase and tumor necrosis factor receptor associated factor 6 (TRAF6) to activate the canonical inhibitor-κB (IκB) kinase (IKK) and degenerate IκBα leading to nuclear factor kappa B (NF-κB) transcriptional activation^11^,^12^. MAPKs has four members in the family, including extracellular signal-regulated kinase1/2 (ERK1/2), extracellular signal- regulated kinase 5 (ERK5), c-Jun N-terminal kinases (JNK) and p38 mitogen- activated protein kinases (p38MAPK)^13^. NF-κB is known as a critical link to inflammatory responses^14^ which has been studied intensively. These cascaded transcriptional reactions finally regulating the releases of inflammatory cytokines and anti-inflammatory factor^15^,^16^. Therefore, the TLR4/NF-κB/MAPKs pathways are considered as one of the main signaling pathways involved in inflammatory response. Hence, inhibition of TLR4, NF-κB and MAPKs signaling cascades is not only a novel idea for treatment of inflammatory and infectious diseases, but also an important target to develop new anti-inflammatory drugs.

Resveratrol (3,4′,5-Trihydroxy-trans-stilbene, RSV; Figure S1) is a polyphenolic phytoalexin which was first isolated from the roots of white hellebore in 1940^17^. RSV has varieties of pharmacological activities, including anticancer^18^, antioxidative^19^, antiviral^20^, immune-regulating^21^ and anti-inflammatory^22^ effects. As RSV is expected to be a new drug candidate for treatment of inflammation, the anti-inflammatory mechanism has always been concerned. It has been reported that RSV could restrain inflammatory effect in liver of old mice by reducing kinds of proinflammatory cytokines^23^, alleviate LPS-induced inflammation in Caco-2 and SW480 human colon cancer cells through inhibiting NF-κB pathway^24^, protect LPS-induced extracellular lipoperoxidation via Myd88-dependent signaling pathway^25^, mitigate lipopolysaccharide- and Aβ-mediated microglial inflammation by inhibiting the TLR4/NF-κB/STAT signaling cascade^12^ and modulate the cytokines-stimulated activation of SAPK/JNK pathway in HT-29 human colon cancer cells^26^. According to these researches, the similar molecular events that involves TLR4, NF-κBp65 and MAPK members (p38MAPK, JNK, ERK1/2, ERK5) affected by RSV have already been separately reported, however, the combined evaluation of all these molecular mechanism is still needed. Therefore, this study was conducted to evaluate the anti-inflammatory effects of RSV in LPS-stimulated rats and elucidate the mechanism through the changes of the signaling cascades of TLR4, NF-κB and MAPK members.

## Results

### RSV inhibited the edema of organs

**Figure 1:**
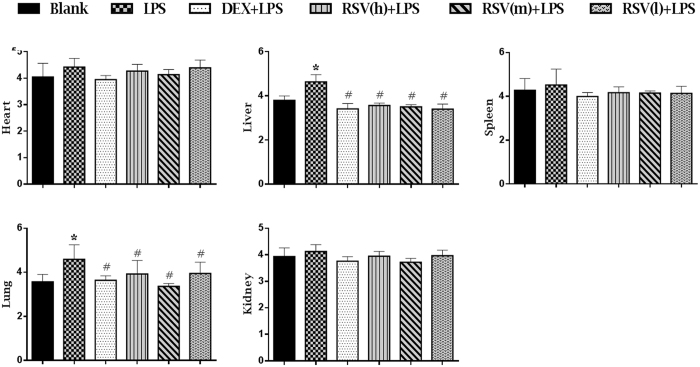
The effects of RSV on viscera wet/dry weight ratios (W/D) induced by LPS. DXM + LPS, RSV(h) + LPS, RSV(m) + LPS and RSV(l) + LPS represent the groups which pretreatment with DXM, high, intermediate and low dose of resveratrol before giving them LPS-stimulus, respectively. The values are presented as means ± standard deviation (10 rats/group). *P < 0.05 vs. Blank control group; ^#^P < 0.05 vs. LPS group.

The W/D ratios of organs were correlated positively with severity of tissue injury^27^. As shown in Fig. 1, the W/D ratios of liver and lung were significantly increased in LPS group compared with those in blank group (P < 0.05), while in groups of pretreatment with DXM and RSV, the W/D ratios were significantly reduced (P < 0.05). The results also showed that the W/D ratios of heart, spleen and kidney in LPS group were slightly higher than other groups.

### RSV could restore the hematological parameters to normal level

The changes of hematological parameters are often associated with physiological changes. As shown in Table S1, we found LPS markedly reduced the production of WBC, RBC, PLT, LYM, GRA, MON and the content of HGB and HCT compared with blank control group (P < 0.01). After pretreatment with DXM and RSV, these hematological parameters were significantly reduced (P < 0.01). No statistical differences in the levels of HCT, MCV, MPV and MCH among all groups were observed.

### RSV inhibited LPS-induced increase in serum biochemistry parameters

The levels of ALT, AST, and ALP in serum are widely used as credible markers on liver damage^28^, and CRE and BUN are useful indicators of renal function^29^. The results showed that the levels of serum biochemistry, including ALP, ALT and AST in LPS group, were significantly increased when compared with those in blank control group (Table S2). After pretreatment with DXM and RSV, these indicators were significantly decreased (P < 0.05). No statistical differences in the levels of BUN, CRE and CHO among all groups were observed.

### RSV alleviated the damages on liver and lung

**Figure 2:**
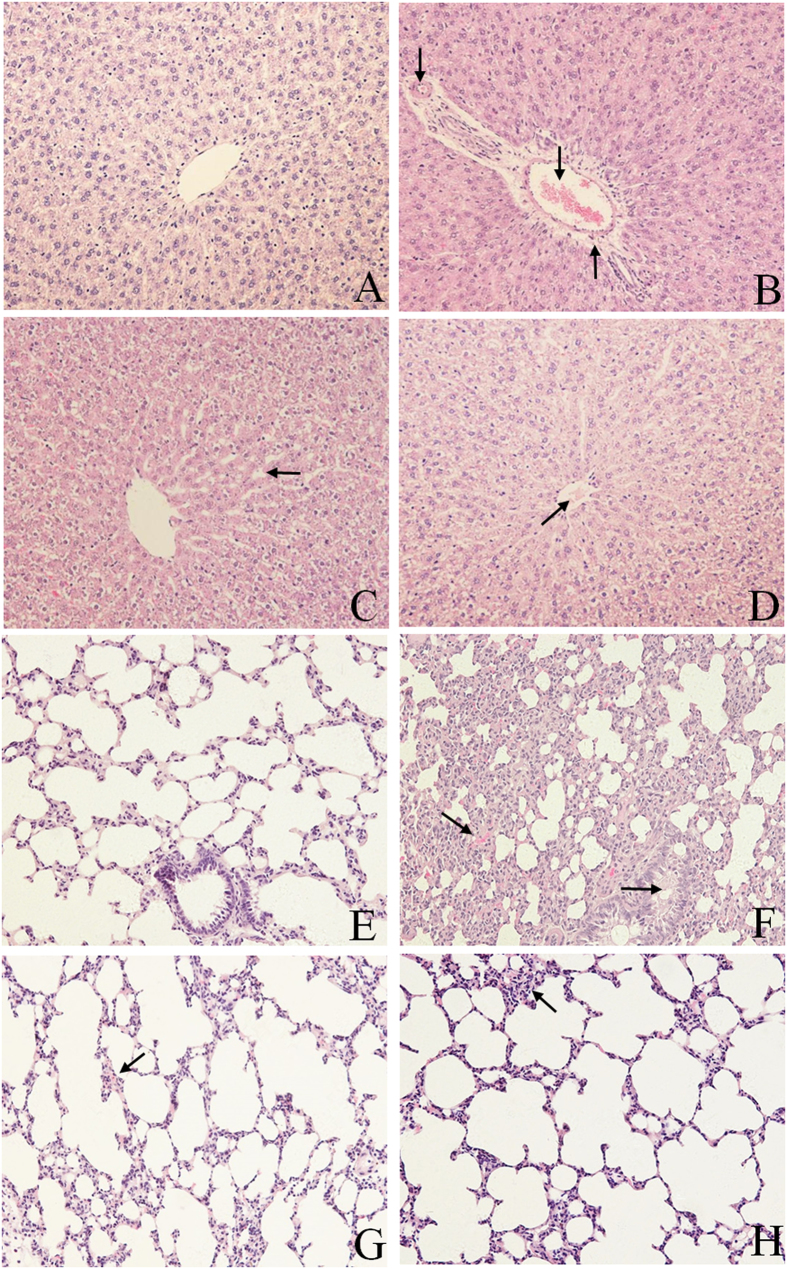
Pathological lesions of liver and lung in different groups. (**A**,**E**) Blank control group; (**B**,**F**) LPS group; (**C**,**G**) DXM-treated group; (**D**,**H**) RSV-treated group. (**A** to **D**) Liver; (↓) in B indicates central veins and interlobular arteries congestion, (↑) in B indicates the blood vessel walls surrounding with lots of inflammatory cells and are shriveling from hepatocytes. (←) in C indicates intervals between hepatic cord slightly widen. 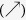 In D indicates the central veins infiltrating with a small amount of seriflux. (**E** to **H**) lung; 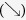 in F indicates alveolar interval seriously thicken and hyperemia, (→) in (**F**) indicates bronchial wall thicken and lumen narrow. 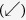 In G indicates alveolar interstitial congesting with some red blood cells. 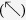 In H indicates visible inflammatory cells increase in alveolar interstitial. Figures in (**A**–**H**) were observed under (HE 200x) optical microscope.

Pathological observation intuitively exhibits the lesions of tissues and organs. Based on the previous results of viscera W/D ratios and serum biochemistry, we conducted the histopathology assessments of the liver and lung of rats. As shown in Fig. 2, normal hepatic structures of liver could be seen in blank control group (Fig. 2A). In LPS group, the central veins and interlobular arteries were seriously congested; the blood vessel walls were surrounded with lots of inflammatory cells and were shriveled from hepatocytes (Fig. 2B); In treated (DXM and RSV) groups, the pathological lesions of liver were significantly alleviated compared with those in LPS group. The intervals between hepatic cord in DXM-treated group were slightly widened (Fig. 2C) and the central vein in RSV-treated group was infiltrated with a small amount of seriflux (Fig. 2D).

In the lung, normal structures of bronchia and alveoli were observed in blank control group (Fig. 2E). In LPS group, alveolar space was seriously thickened and hyperemia; the bronchial wall was thickened and the lumen was narrowed (Fig. 2F). After pretreatment with DXM and RSV, the pathological lesions of lung were slight. The alveolar space in DXM-treated group was congested with red blood cells (Fig. 2G), and visible inflammatory cells in alveolar space were increased in RSV-treated group (Fig. 2H).

### RSV suppressed the production of proinflammatory mediators and cytokines, and increased the release of anti-inflammatory factor

**Figure 3:**
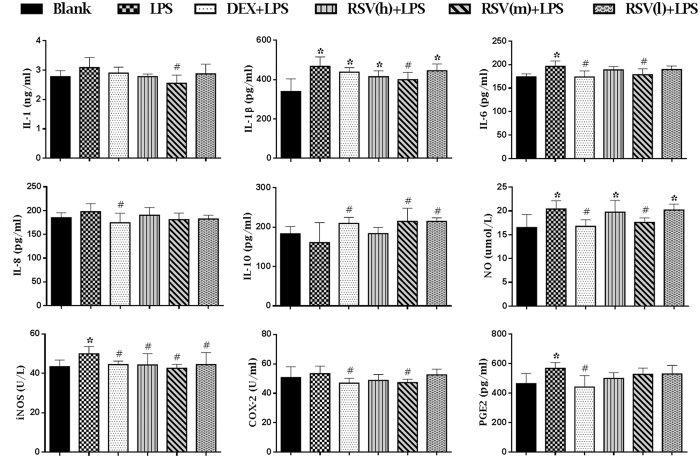
The effects of RSV on the content of inflammatory cytokines and proinflammatory mediators induced by LPS were detected by ELISA. DXM + LPS, RSV(h) + LPS, RSV(m) + LPS and RSV(l) + LPS represent the groups which pretreatment with DXM, high, intermediate and low dose of resveratrol before giving them LPS-stimulus, respectively. The values are presented as means ± standard deviation (10 rats/group). *P < 0.05 vs. Blank control group; ^#^P < 0.05 vs. LPS group.

Inflammatory mediator is a kind of chemical medium with a strong biological activity, which not only can accommodate the release of it-self, but also can activate other mediators, producing a series of cascade amplification reactions and making further development of inflammation^30^. The results of cytokine concentrations in serum were shown in Fig. 3. Compared with blank control group, the productions of proinflammatory mediators and cytokines, including IL-1β, IL-6, NO, iNOS and PGE_2_, were significantly increased in LPS group (P < 0.05). On the contrary, these proinflammatory mediators and cytokines in treated (DXM and RSV) groups were significantly decreased (P < 0.05), especially in intermediate dose of RSV group. Though there were no statistical differences between blank control group and LPS group on the expression of proinflammatory cytokines and mediator (IL-1, IL-8 and COX-2) and anti- inflammatory factor (IL-10), the IL-1, IL-8 and COX-2 were relatively increased and the IL-10 was relatively decreased in LPS group. Similarly, there were no statistical differences between LPS group and RSV-treated groups on the expression of IL-8 and PGE2, but they all relatively decreased in RSV-treated groups, especially in the groups of intermediate and low doses. Compared with LPS group, the IL-10 was significantly increased in treated (DXM and RSV) groups (P < 0.05).

### RSV restrained LPS-induced increase of mRNA levels of TLR4, NF-κBp65 and MAPK members in liver and lung

**Figure 4:**
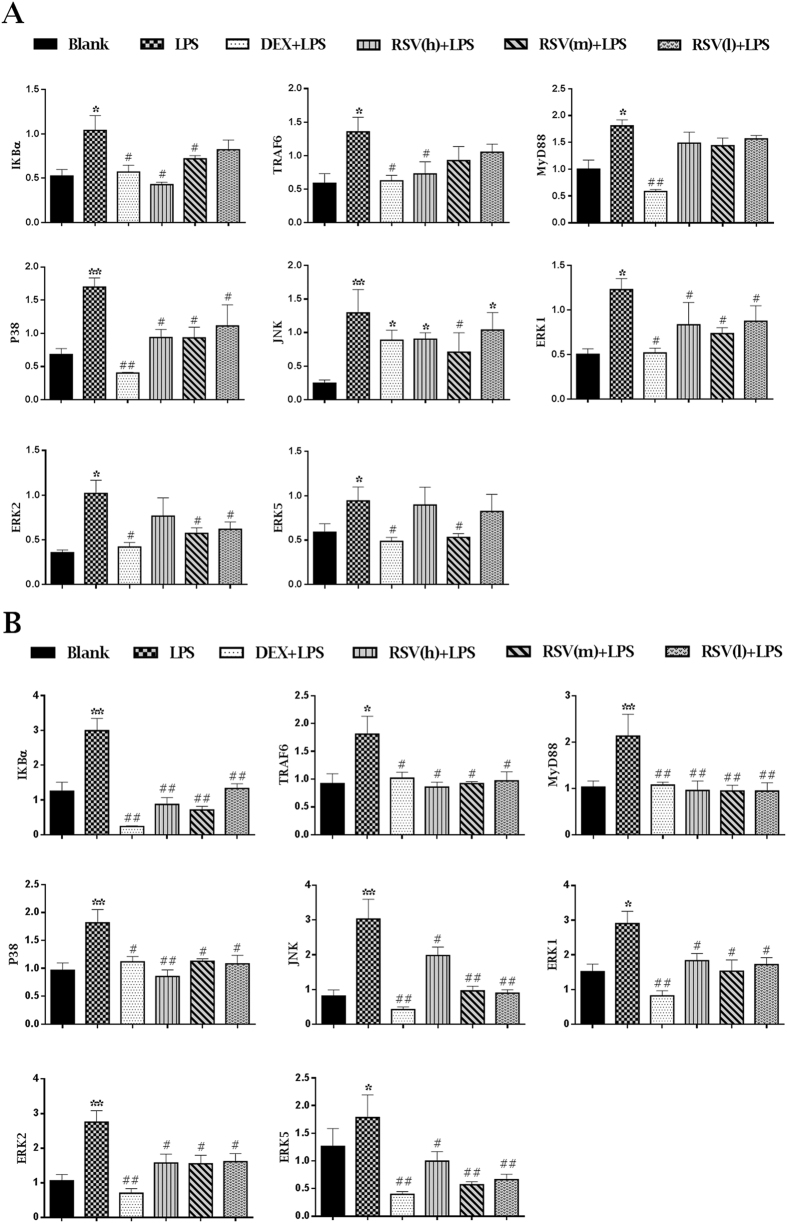
The mRNA levels of MyD88, TRAF6, IKBα, P38, JNK, ERK1, ERK2 and ERK5 in liver (**A**) and lung (**B**) assessed by RT-PCR. DXM + LPS, RSV(h) + LPS, RSV(m) + LPS and RSV(l) + LPS represent the groups which pretreatment with DXM, high, intermediate and low dose of resveratrol before giving them LPS-stimulus, respectively. The values are presented as means ± standard deviation (10 rats/group). *P < 0.05, **P < 0.01 vs. Blank control group; ^#^P < 0.05, ^##^P < 0.01 vs. LPS group.

The mRNA levels of related genes in liver and lung assessed by RT-PCR were shown in Fig. 4A and B. Compared with blank control group, the mRNA levels in LPS group involving MyD88, TRAF6, IĸBα, p38MAPK, JNK, ERK1, ERK2 and ERK5 both in liver and lung were significantly up-regulated. Pretreatment with DXM and RSV, the mRNA levels of these genes in lung and liver were down-regulated.

### RSV attenuated the expression of protein levels including TLR4, NF-κBp65 and MAPK members in liver and lung

**Figure 5:**
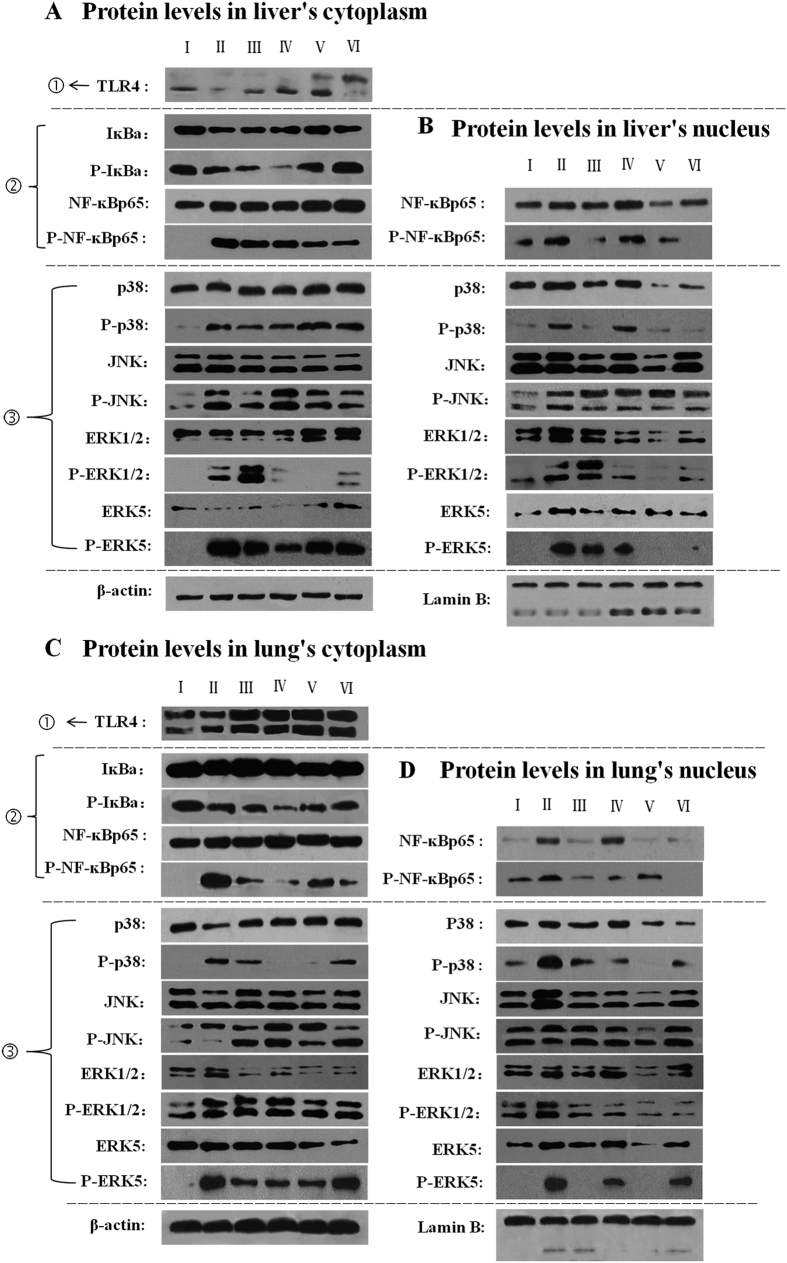
Protein and phosphorylated protein levels of TLR4, IĸBα, NF-κBp65, p38, JNK, ERK1/2 and ERK5 in liver and lung were preformed by Western blotting. In each rank, the dynamic changes of proteins and phosphorylated proteins from tissue’s cytoplasm to nucleus could be seen. ①: The expression of related protein in TLR4 signaling pathway; ②: The expression of related proteins and phosphorylated proteins in NF-κBp65 signaling pathway; ③: The expression of related proteins and phosphorylated proteins in MAPKs signaling pathway. (**A**,**B**) Protein and phosphorylated protein levels in liver’s cytoplasm and nucleus, respectively; (**C**,**D**) protein and phosphorylated protein levels in lung’s cytoplasm and nucleus, respectively. I, II, III, IV, V and VI represent the groups of blank control, LPS, DXM-treated, high dose, intermediate dose and low dose of RSV-treated group, respectively.

Based on the results of mRNA levels, the western blotting analysis of liver and lung on TLR4, NF-ĸBp65 and MAPK members were performed. As shown in Fig. 5, we found that, in the liver’s cytoplasm, the protein level of TLR4 in TLR4 signaling pathway, the IĸBα in NF-ĸBp65 signaling pathway and the p38MAPK, ERK1/2 and ERK5 in MAPKs signaling pathway in LPS group were lower than those in blank control group, but the levels of these proteins in intermediate and low doses of RSV-treated groups were higher expressed than those in LPS group (Fig. 5A; Figure S2a,b and d). In liver’s nucleus, the expression of NF-ĸBp65, p38MAPK, ERK1/2 and ERK5 as well as their phosphorylated proteins in LPS group were higher than those in blank control group. Whereas, the levels of these proteins and phosphorylated proteins in intermediate and low doses of RSV-treated groups were lower than those in LPS group (Fig. 5B; Figure S2c and e). The protein levels in lung’s cytoplasm were shown in Fig. 5C. The levels of TLR4, IĸBα, p38MAPK and JNK in LPS group were lower than those in blank control group. However, the levels of these proteins expression in intermediate and low doses of RSV-treated groups were higher than those in LPS group (Figure S2f,g and i). In the lung’s nucleus, the protein levels of NF-ĸBp65, p38MAPK, JNK, ERK1/2 and ERK5 as well as their phosphorylated protein levels in LPS group were all obviously higher than those in blank control group. But these proteins and their phosphorylated proteins in all pretreated (DXM and RSV) groups were lower than those in LPS group (Fig. 5D; Figure S2h and j). The protein levels of P-NF-κBp65, P-IĸBα, P-p38MAPK, P-JNK, P-ERK1/2 and P-ERK5 in liver’s and lung’s cytoplasm were different from each other, but each phosphorylated protein in blank control group showed no expression or relatively small amount expression in comparison with those in LPS group except the P-IĸBα. The expression of P-IĸBα in LPS group was lower than that in blank control group (Fig. 5A and C; Figure S2b and g).

## Discussion

LPS, as a potent cytotoxic inducer of inflammation, could cause lots of damages to organs, including heart, liver, spleen, lung and kidney^31^,^32^,^33^,^34^,^35^,^36^. However, which organ would be affected when intraperitoneal injection with LPS and how’s RSV protecting these organs from inflammation remain disputable. In this study, the results showed that the W/D ratio only in liver and lung were significantly increased in LPS group (Fig. 1), indicating that intraperitoneal injection with LPS could cause damages to liver and lung, but no obvious side effects on heart, spleen and kidney. Pretreatment with RSV could effectively protect rats against LPS-induced acute inflammation. Based on this result, we conducted clinical pathology determination and histopathological examination for further validation. The results of hematology indexes showed that the levels of WBC, RBC, PLT, HGB, LYM, GRA and MON in LPS group were significantly reduced, and pretreatment with RSV could restore these parameters to normal levels (Table S1). These results revealed that LPS could cause alteration in hematological parameters and RSV could protect the blood circulatory system, which was similar to the previous report^37^. According to the changes of biochemical indicators (Table S2), it could be concluded that intraperitoneal injection with LPS could cause damages to liver rather than kidney, which was accordant with the findings in W/D ratios. Pretreatment with RSV could inhibit LPS-induced increase in AST, ALT and ALP, suggesting RSV could protect the liver from the damages induced by LPS, which was consistent with the mechanism proposed by Zhong^38^. The histopathological examination results showed that LPS could cause severe lesions in liver and lung, and RSV could obviously alleviate the lesions (Fig. 2). This result suggested that RSV had a protective effect on LPS-induced tissue injury, which was consistent with other reports^23^,^35^,^39^.

The activated reaction of signaling pathways can regulate the release of inflammatory cytokines^15^ which play a critical role in inflammatory response^40^. Accumulating evidences indicate that interleukins, including IL-1, IL-1β, IL-6, IL-8, have strong influences on inflammatory responses. They serve as markers in LPS- induced acute inflammation^41^,^42^,^43^. IL-10 is often associated with suppression or reduction of inflammatory responses^44^. Our results showed that intraperitoneal injection with LPS could elevate the production of proinflammatory cytokines and reduce the release of anti-inflammatory factor (IL-10). Pretreatment with RSV could attenuate the production of IL-1, IL-1β and IL-6, but increase the release of IL-10, especially in intermediate dose of RSV (Fig. 3). These results were consistent with the report that treatment with RSV could significantly inhibit the expressions of NO, IL-1β, IL-6 and TNF-α in LPS-induced RAW264.7 and peritoneal macrophage cells^45^. NO is a pivotal molecule in many body functions, but its overproduction may lead to inflammation and autoimmune disorders^46^. iNOS is one of the key enzymes generating NO from arginine in inflammatory reaction. Therefore, NO and iNOS are important mediators in acute and chronic inflammatory processes^47^. PGE_2_ is the main metabolite of arachidonic acid which produced by the inducible enzyme COX-2 and is also important for inflammation diagnosis^48^. Therefore, inhibition of the production of iNOS, NO, COX-2 and PGE_2_ might have potential therapeutic effects for preventing inflammatory diseases. Our results showed the contents of NO, iNOS and PGE_2_ were significantly increased after LPS injection, and pretreatment with RSV could decrease the contents of these proinflammatory mediators, especially in intermediate dose of RSV (Fig. 3). All these results illustrated that RSV could mitigate inflammatory reactions by decreasing the production of proinflammatory mediators and cytokines and increasing the expression of anti-inflammatory factor. The phenomenon that intermediate dose of RSV showed higher effect than the highest dose in several pro-inflammatory mediators, which was similar with previous researches^21^,^24^,^26^. The possible reason may be due to RSV exhibiting a two-ways- regulating effect and the biotransformation at high dose of RSV making the most ineffective activity. The production of biotransformation may be caused by, for example, UDP-glucuronosyl-transferases UGT1A7 and UGT1A10 which are both expressed in human gastrointestinal tract and could catalyse RSV glucuronidation or sulphate conjugation^49^,^50^. But this issue still needs future investigation. Moreover, the result showed the IL-1β and NO in high and low doses of RSV-treated groups were significant differences with those in blank control group, while other pro-inflammatory mediators, including IL-1, IL-6, IL-8, IL-10, iNOS, COX-2 and PGE2 in all RSV-treated groups showed no significant differences. This result was coincident with previous results that there were no significant differences between normal group and RSV-treated groups (The group only administrated with RSV and the group supplemented with RSV after stimulation)^39^,^51^,^52^,^53^, suggesting that at normal state, whether administration with RSV showed no significant differences on the evaluation of anti-inflammatory activity. Furthermore, our results also showed that the contents of NO and PGE2 were almost corresponding with the production of iNOS and COX-2, respectively, which further substantiated the rationale that iNOS is one of the key enzymes generating NO and PGE_2_ is produced by the inducible enzyme COX-2.

In recent years, several researches about anti-inflammatory mechanism of RSV have been reported^23^,^24^,^25^,^26^. However, these researches mainly confined to just one or two signaling pathways and they were mainly studied just *in vitro*. In addition, TLR4, NF-κB and MAPK signaling pathways are known as pivotal links to inflammatory responses^54^,^55^,^56^. Therefore, we established LPS-stimulated rats to explore the cascades of molecular events of RSV inhibiting TLR4, NF-κBp65 and MAPKs (ERK1/2, ERK5, JNK, p38MAPK) signaling pathways *in vivo*. MyD88 is one of the main adaptor proteins for TLR4 to promote signal transduction^8^. Thus, high mRNA level of MyD88 may indicate that the TLR4 pathway is activated. Meanwhile, MyD88- dependent pathway may activate MAPK and NF-κB pathways via TRAF6 and IκBα intermediary molecules^9^,^12^,^25^. The TRAF6 and IκBα molecules are necessary mediators for activation of MAPK and NF-κB signaling pathways. Only when NF-kB signaling pathway is stimulated by external stimulus, the IκBα protein is activated and rapidly phosphorylated by IkB kinase. In our test, the NF-kB signaling pathway is stimulated by LPS, thus leading to the activation of IκBα protein and the temporary increasing of IκBα mRNA level. Therefore, the data displayed that after LPS injection, the mRNA levels of MyD88, TRAF6, IĸBα, p38MAPK, JNK, ERK1, ERK2 and ERK5 both in rats’ liver and lung were significantly up-regulated. After pretreatment with RSV, the mRNA levels of these genes in rats’ liver and lung were all down-regulated (Fig. 4A and B). These results suggested that LPS-stimulation motivated the generation of inflammatory reaction that promoted the combination of MyD88 with TRL4, then activated NF-κB, p38MAPK, JNK, ERK1/2 and ERK5 signaling pathways through recruiting TRAF6 and phosphorylating IĸBα. RSV exerted an anti-inflammatory and protective effects through inhibition of the combination of MyD88 with TRL4 and further suppressing the cascades activation of NF-κB and MAPKs (ERK1/2, ERK5, JNK, p38MAPK) signaling pathways. These findings were similar with the anti-inflammatory effects of cordycepin induced by LPS^57^.

To further validate the results of mRNA levels, the western blotting assay was carried out. It has been reported that all of the toll-like receptors are the first-type transmembrane proteins including TLR4, which is a crucial factor in triggering the inflammatory response by recognizing the associated ligands^7^. Then, the TLR4-mediated signal pathway activates the related signaling pathways, such as NF-kB and MAPK, which could regulate the release of proinflammatory mediators and cytokines^39^. At resting-state, NF-kBp65 is combing with the inhibitory protein IκB which can make NF-kB retained in the cytoplasm in an inactive forms of dimers. Once NF-kB signaling pathway is stimulated by external stimulus, concomitant IκB protein rapidly phosphorylated in response and then subsequently degraded by the proteosomal pathway. Finally, these reactions lead to the activation of NF-kB signaling pathway. And at the same time, NF-kB in cytoplasm is transferred into the nucleus^58^. p38MAPK is a relay of many signals which exists both in cytoplasm and nucleus, once be stimulated, it would be activated through a series of intermediate reactions and the p38MAPK in cytoplasm is ultimately transferred into the nucleus^59^,^60^. The JNK and ERK1/2 pathways are also important to inflammatory response and both of them exist in cytoplasm under normal state, after stimulation, JNK and ERK1/2 in cytoplasm are accumulated into the nucleus^61^,^62^. ERK5 is the only member of MAPK which can be activated not only by mitogens but also by stress conditions. Once it was activated, ERK5 in cytoplasm is transferred into the nucleus^63^. Moreover, ERK5 exhibits a long C-terminal regulatory domain exerting trans-activating transcriptional property that distinguishes it from other MAPK members^64^. All theories above show that the activation of a signaling pathway is a dynamic process. Once the pathways are activated, the related proteins will be transferred from the cytoplasm into the nucleus. However, the TLR4 and IκBα only change in the cytoplasm.

Our results exhibited that the protein levels of TLR4, IĸBα, p38MAPK, ERK1/2 and ERK5 in liver’s cytoplasm were lowly expressed under the stimulus of LPS, but high expression of NF-ĸBp65, p38MAPK, ERK1/2 and ERK5 as well as their phosphorylated proteins in liver’s nucleus (Fig. 5A and B; Figure S2a–e). Similar with liver, the levels of TLR4, IĸBα, p38MAPK and JNK in lung’s cytoplasm were also lowly expressed after injection with LPS, but in the lung’s nucleus, the expression of NF-ĸBp65, p38MAPK, JNK, ERK1/2 and ERK5 as well as their phosphorylated proteins were all highly expressed (Fig. 5C and D; Figure S2f–j). These results illustrated that different organs behaved differently in the inflammatory response to LPS stimulus, thus, different pathways have been activated. Taking the liver as an example, LPS stimulus activated the pathways of TLR4, NF-κBP65, ERK1/2 and ERK5, and then led to phosphorylated of IκBα, NF-κBP65, ERK1/2 and ERK5 immediately. Meanwhile, the NF-κBP65, ERK1/2 and ERK5 in liver’s cytoplasm were transferred into nucleus, finally led to low expressions of TLR4, IκBα, NF-κBP65, ERK1/2 and ERK5 in liver’s cytoplasm, but high expressions of NF-κBP65, ERK1/2, ERK5 and their phosphorylated proteins in nucleus. These results were consistent with the report that NF-κBp65 was normally sequestered in the cytoplasm but strongly accumulated in nuclear after stimulating with LPS in RAW 264.7 cells^57^. Our results also showed that, after pretreatment with RSV, the corresponding proteins in liver’s and lung’s cytoplasm were highly expressed, but low production of related proteins and phosphorylated proteins in their nucleus (Fig. 5A to D; Figure S2a–j). These results suggested that RSV could inhibit the inflammatory response in liver and lung by decreasing the transfer reaction of signals from cytoplasm to nucleus through attenuating the activation of relevant signal pathways. These results were similar with the report that RSV could inhibit PTEN/Akt pathway in prostate cancer cells by regulating MTA1/HDAC protein levels in cytoplasm and nucleus^18^. We also found an identical trend of the phosphorylated protein levels in liver’s and lung’s cytoplasm (Fig. 5A and C; Figure S2b,d,g and i), that was although the protein levels of P-NF-κBp65, P-p38MAPK, P-JNK, P-ERK1/2 and P-ERK5 in liver’s cytoplasm was different from which in lung’s cytoplasm, both in liver’s and lung’s cytoplasm, the levels of these phosphorylated proteins in blank control group showed no expression or relatively small amount expression than those in LPS group. However, the P-IĸBα level in LPS group was lower than that in blank control group, which may due to the inflammatory reaction only in LPS-stimulated group. Therefore, the low content of P-IĸBα in LPS group attributed to its rapid degradation after stimulation. Despite western blotting is widely used in many mechanism researches, most of them are implemented on the basis of total protein and hardly on the cytoplasm and nucleus proteins, respectivley^12^,^39^,^45^. At present, the expressions of proteins in tissue’s cytoplasm and nucleus as well as phosphorylated proteins in tissue’s nucleus could be speculated, but the changes of phosphorylated proteins in tissue’s cytoplasm remains incomprehensive and controversial, which needs further study.

**Figure 6:**
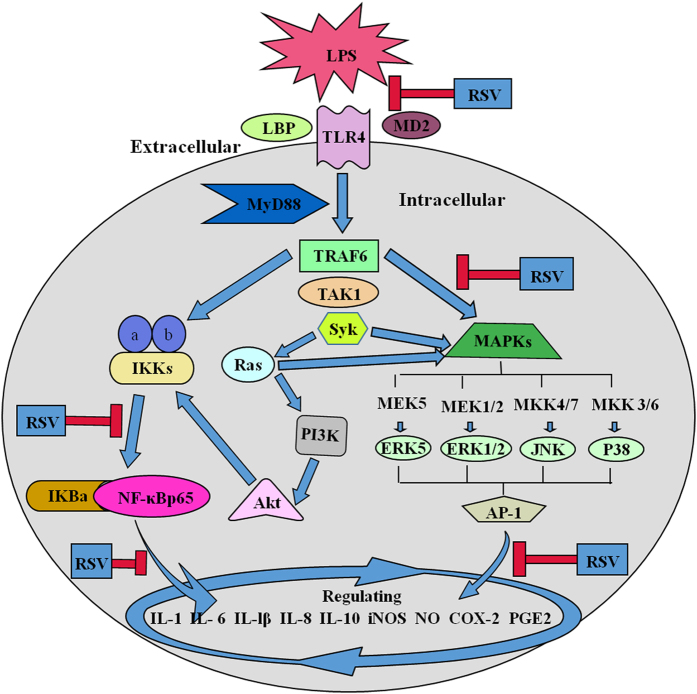
Schematic presentation of possible signaling cascades of RSV in suppression of the LPS-induced inflammatory response. Blue arrows indicate progressive courses of inflammatory response induced by LPS. Red “inverted-T” symbols indicate the possible inhibitory effects of RSV. Key abbreviations: LPS, lipopolysaccharide; RSV, resveratrol; TLR4, Toll-like receptor 4; MyD88, myeloid differentiation factor 88; TRAF6, tumor necrosis factor receptor associated factor 6; MAPKs, mitogen-activated protein kinases; IκBα, inhibitor kappa B alpha; IKK, inhibitor-κB kinase; NF-κBp65, nuclear factor kappa B 3; IL, interleukin; NO, nitric oxide; iNOS, inducible nitric oxide synthase; COX-2, cyclooxygenase-2; PGE2, prostaglandin E2.

In summary, we reported herein the anti-inflammatory effects and molecular cascades of RSV in LPS-stimulated rats. Our results demonstrated that LPS could cause inflammatory damages to liver and lung. Pretreatment with RSV could significantly reduce the LPS-induced lesions through inactivating corresponding genes at the transcriptional levels and related proteins at the translational levels. The anti-inflammatory mechanisms of RSV were summarized in Fig. 6, which attributed to regulating the expressions of corresponding proinflammatory and anti- inflammatory mediators and cytokines by inhibition of the signaling cascades of TLR4, NF-κBp65 and MAPK (p38MAPK, JNK, ERK1/2, ERK5).

## Materials and Methods

### Animal and treatment

60 healthy Sprague-Dawley (SD) rats (containing equal numbers of male and female) weighing 180 ± 20 g were purchased from Chengdu Dossy Experimental Animals Co., Ltd. [License No. SCXK (Sichuan) 2015–23]. The rats were randomly divided into six groups, including blank control group (physiological saline), model group (LPS, 100 μg/kg. *Escherichia coli* 055:B5), RSV-treated groups (high, intermediate and low doses of RSV are 30, 10 and 3 mg/kg, respectively) and dexamethasone-treated group (DXM-treated, 40 μg/kg). The RSV-treated groups were orally pretreated with different dose of RSV; the blank control group and LPS group were orally administrated with an equal volume of physiological saline. Each group was treated for 4 days except the DXM-treated group, which was intraperitoneal injection with DXM only on the fourth day. All groups were intraperitoneal injection with 100 μg/kg LPS at 30 min after the last administration.

### Ethics Statement

All methods and experimental protocols were conducted under the approved guidelines of Sichuan Agriculture University (Chengdu, China) and approved by the ethical committee of the Laboratory Animals Care (Chengdu, China).

### Viscera wet/dry weight ratio

At 8 h after the LPS administration, all animals were euthanized under ether anesthesia and were subjected to a full necropsy. Heart, liver, spleen, lung, kidney were procured and weighed wet immediately, and then they were been put in an oven at a constant temperature of 80°C. After 48 h, each viscera was weighted again and the wet/dry weight (W/D) ratio was calculated.


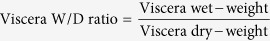


### Hematological examination

The blood samples about 0.5 mL of each animal for hematology assessment were collected in tubes containing EDTA. The hematological parameters including white blood count (WBC), red blood cell count (RBC), hemoglobin concentration (HGB), platelet (PLT), granulocyte (GRA), lymphocyte count (LYM), the average platelet volume (MPV), hematokrit (HCT), mean corpuscular volume (MCV), mean corpuscular hemoglobin (MCH) and monocytes (MON) were measured, respectively.

### Serum biochemistry examination

Approximately 1 mL blood sample of each animal was collected into a tube with no preservative, and after coagulation, serum was separated by centrifugation and used for blood biochemical assessments. The biochemical parameters including alanine aminotransferase (ALT), aspartate aminotransferase (AST), alkaline phosphatase (ALP), urea nitrogen (BUN), creatinine (CRE) and total cholesterol (CHO) were been measured, respectively.

### Histopathological examination

The organs used for histopathological examination were immediately preserved in 4% paraformaldehyde after dissection to avoid drying. They were dehydrated with different concentrations of ethanol, and then enclosed in paraffin. The paraffin section were stained by hematoxylin-eosin (HE) and observed under optical microscope.

### Cytokine assays

The blood samples used for cytokine examination were collected into another non-preservative tube. The serum was separated by centrifugation at 3000 rpm for 10 min after coagulation. The concentrations of Interleukin-1(IL-1), IL-1β, IL-2, IL-6, IL-8, IL-10, cyclooxygenase-2 (COX-2), prostaglandin E2 (PGE2), inducible nitric oxide synthase (iNOS) and nitric oxide (NO) in the serum were measured followed by the guidance of R&D rats ELISA kits (USA).

### Real-time polymerase chain reaction assay

In order to reveal the molecular cascades, the determination of related genes’ mRNA levels was necessary. Briefly, total RNA from liver and lung were extracted by TRIzol reagent (No. 15596026, Invitrogen) according to the manufacturer's protocol. Then, RNA quality was determined by measuring the 260/280 ratio (>2.0). RNA (1 μg) of each tissue was reverse-transcribed by Revert Aid first-strand cDNA synthesis kit (No. K1621, Thermo Scientific). cDNA (1 μl) of each tissue’s sample was used for RT-PCR with SYBR Green Supermix kit (No. 1708882, Bio-Rad). According to previous reports^65^,^66^, the levels of mRNA, expressed as relative mRNA levels compared with control, were calculated three times in duplicate after normalization to β-actin. Sequences for primers were design by Oligo 7 software (Table S3). Primers were synthesized by Liuhe genomics technology co., LTD (Beijing, China).

### Western blotting assay

To clearly illuminate whether a signaling pathway was activated, the cytoplasmic protein and nucleoprotein were extracted for evaluation, respectively, rather than total protein in this test. As carried out previously^67^, liver and lung were collected and washed with cold PBS, and then were lysed with lysis buffer containning 1 mM inhibitor PMSF (No. AR1178, Boster). The cytoplasmic protein and nucleoprotein of liver and lung were extracted by the subcellular structure of cytoplasm protein and nucleoprotein extracted kit (No. AR0106, Boster), respectively. The concentrations of cytoplasmic protein and nucleoprotein were determined by the BCA protein assay kit (No. AR0146, Boster). Then, each sample was subsequently denatured with 4× dualcolor protein loading buffer at 100°C for 5 min (No. AR1142, Boster). Equivalent amounts of protein (30 μg) from each sample were separated by sodium dodecyl sulfate polyacrylamide gel electrophoresis (SDS-PAGE) and subsequently transferred to polyvinylidene fluoride (PVDF) membranes (Millipore, USA). Protein blots were blocked with 5% skim milk or 5% BSA at room temperature for 1 h and then were incubated with primary antibodies against TLR4, IκBα, NF-κBp65, P-NF-κBp65, p38MAPK, P-p38MAPK, JNK, P-JNK, p44/42, P-p44/42, ERK5, P-ERK5 (CST, USA) overnight at 4°C. After washing three times with TBST [20 mM Tris–HCl (pH 7.4), 100 mM NaCl and 0.1% (w/v) Tween 20] buffer and then protein blots were exposed to the relative peroxidase-conjugated secondary antibody (Goat anti-mouse or goat anti-rabbit, CST, USA) for 1 h at room temperature. Immunoreactive bands were visualized with chemiluminescence (ECL prime, Pierce Chemical, USA) using standard X-ray film (Kodak X-Omat AR) and quantified relative to β-actin or Lamin B (CST, USA) using National Institutes of Health Image J software^18^,^68^.

### Statistical analysis

The data are presented as the mean ± standard deviation, and all of the statistical analyses were performed using SPSS 17.0 statistical software. For histograms used in viscera wet/dry weight ratio, cytokine examination, mRNA levels expression and protein’s mean density expression were plotted by GraphPad Prism 6 software. Statistical significance of the data from the control and experimental groups was compared by one way analysis of variance (ANOVA) and the Student-Newman-Keuls test^21^. P < 0.05 was considered statistically significant.

## Additional Information

**How to cite this article:** Wang, G. *et al*. Resveratrol mitigates lipopolysaccharide-mediated acute inflammation in rats by inhibiting the TLR4/NF-κBp65/MAPKs signaling cascade. *Sci. Rep.* **7**, 45006; doi: 10.1038/srep45006 (2017).

**Publisher's note:** Springer Nature remains neutral with regard to jurisdictional claims in published maps and institutional affiliations.

## Supplementary Material

Supplementary Information
