# Supplementary material for: Resveratrol mitigates lipopolysaccharide-mediated acute inflammation in rats by inhibiting the TLR4/NF-κBp65/MAPKs signaling cascade
Source: Sci Rep. 2017 Mar 21;7:45006. doi: 10.1038/srep45006 (PMC5359552; doi:10.1038/srep45006)
Supplement: Supplementary Information [file srep45006-s1.pdf]

Resveratrol mitigates lipopolysaccharide-mediated acute inflammation in rats by inhibiting the TLR4/NF-κBp65/MAPKs signaling cascade

Guangxi Wang [a,1], Zhiqiang Hu [b,1], Qiuting Fu [a,1], Xu Song [a,1], Qiankun Cui [a], Renyong Jia[b*], Yuanfeng Zou[a] , Changliang He[a], Lixia Li [a], Zhongqiong Yin[a, 1*].

aNatural Medicine Research Center, College of Veterinary Medicine, Sichuan Agricultural University , Chengdu, 611130, China

b *Key laboratory of Animal Disease and Human Health of Sichuan Province, Sichuan Agricultural University, Chengdu 611130,China*

[1] These authors contribute equally to this work and should be considered as the first author.

* Corresponding author:

yinzhongqiong

Postal address:Natural Medicine Research Center, College of Veterinary Medicine, Sichuan Agricultural University, Chengdu, 611130, PR China

Tel: +86 835 2885614; Fax: +86 835 2885302

E-mail address: yinzhongq@163.com (Z.Q. Yin).

Ren-Yong Jia, E-mail address: cqrc_jry@163.com

Supplementary Table S1 The effects of RSV on hematologic determination induced by LPS.

Items	Blank control	LPS	DXM+LPS	RSV(h)+LPS	RSV(m)+LPS	RSV(l)+LPS
WBC (10^9/L)	11.200±3.450	3.350±1.083**	11.767±2.665##	8.700±3.431##	9.650±4.003##	7.317±2.515##
RBC (10^{12}/L)	6.642±1.255	4.645±1.273*	6.908±1.307#	6.913±1.659#	7.037±1.071#	6.533±0.665#
HGB	132.50±27.13	94.17±30.22**	139.17±27.24##	139.00±33.40##	140.83±20.90##	130.83±13.26##
PLT(10^9/L)	619.67±182.58	325.50±199.10*	825.83±304.73##	778.67±357.75##	882.00±341.51##	552.67±206.14#
LYM(10^9/L)	7.833±2.338	2.850±1.432**	8.650±1.699##	6.300±2.983##	7.367±3.222##	5.733±2.181##
GRA (10^9/L)	3.017±1.070	0.483±0.232**	2.733±0.987##	2.100±0.890##	2.017±0.801##	1.367±0.314#
MON(10^9/L)	0.350±0.105	0.017±0.041**	0.383±0.117##	0.300±0.110##	0.267±0.103##	0.217±0.075##
HCT	40.017±7.841	32.950±10.840	41.633±8.057	41.817±10.030	42.117±5.918	39.467±3.990
MCV	60.267±2.859	61.517±3.399	60.283±1.663	60.550±1.992	60.067±1.890	60.533±1.496
MPV(fl)	5.817±0.366	6.100±0.167	6.250±0.472	6.183±0.436	6.567±0.393	6.267±0.308
MCH	19.867±0.961	20.000±1.287	20.067±0.480	20.067±0.706	20.000±0.648	19.983±0.553

WBC: white blood cell count; RBC: red blood cell count; HGB: hemoglobin concentration; PLT: platelet; GRA: granulocyte; LYM: lymphocyte count; MPV: the average platelet volume; HCT: hematokrit; MCV: mean corpuscular volume; MCH: mean corpuscular hemoglobin and MON: monocytes. DXM+LPS, RSV(h)+LPS, RSV(m)+LPS and RSV(l)+LPS represent the groups which pretreatment with DXM, high, intermediate and low dose of resveratrol before giving them LPS-stimulus, respectively. The values are presented as means±standard deviation (10 rats /group). * P<0.05, ** P<0.01 vs. Blank control group; # P<0.05, ## P<0.01 vs. LPS group.

Supplementary Table S2 The effects of RSV on blood biochemical indicators induced by LPS.

Groups	ALT(U/L)	AST(U/L)	ALP(U/L)	BUN(mmol/L)	CRE(μmol/L)	CHO(mmol/L)
Blank control	48.17±6.40	235.00±15.54	238.83±73.81	5.5350±1.3242	67.83±4.36	2.1083±0.1385
LPS	55.33±2.42*	286.67±38.59*	366.67±119.04*	4.9450±0.6137	64.50±2.26	1.6900±0.3309
DXM+LPS	49.50±5.32#	214.67±37.40#	263.17±86.30#	5.9517±1.2760	57.67±11.17	2.3050±1.0893
RSV(h)+LPS	43.83±9.56#	178.83±69.33##	213.00±56.89#	4.1967±0.4699	63.67±9.73	1.9583±0.4853
RSV(m)+LPS	49.67±11.73#	215.67±50.03#	221.83±70.11#	4.6383±0.9238	64.67±4.93	1.8483±0.2143
RSV(l)+LPS	43.50±13.13#	187.17±60.76##	242.17±186.14#	4.9200±1.1313	61.17±9.62	1.9933±0.2100

ALT: alanine aminotransferase; AST: aspartate transaminase; ALP: alkaline phosphatase; BUN: blood urea nitrogen; CRE: Creatinine; GLU: glucose; CHO: total cholesterol. DXM+LPS, RSV(h)+LPS, RSV(m)+LPS and RSV(l)+LPS represent the groups which pretreatment with DXM, high, intermediate and low dose of resveratrol before giving them LPS-stimulus, respectively. The values are presented as means ± standard deviation (10 rats/group). * P<0.05, ** P<0.01 vs. Blank control group; # P<0.05, ## P<0.01 vs. LPS group.

Supplementary Table S3 Primer sequences used in RT-PCR analysis.

Primer	Primer sequences Fragment size (5′>3′) (forward/reversed)	Length(bp)
MyD88	F-5′TTGCTAGCCTTGTTAGACCGT3’<br>R-5′CTCCTGTTTCTGCTGGTTGCG3’	99
TRAF6	F-5′ACCATCAAATCCGGGAGC3’<br>R-5′TGCCAATCTTCCAAATGTAAATGC3’	120
IκBα	F-5′CCACCAACTACAACGGCCACAC3’<br>R-5′TTGACATCAGCACCCAAAGTCAC3’	97
P38	F-5′AGTGGCTGACCCTTATGAC3’<br>R-5′CACAGTGAAGTGGGATGGA3’	168
ERK5	F-5′GGTTGCCTGGTTCTGGT3’<br>R-5′TTGGACAGTTGCTGGGT3’	136
ERK1	F-5’CTACACGCAGCTGCAGTACATC-3’<br>R-5’GTGCGCTGACAGTAGGTTTGA-3’	132
ERK2	F-5’GGCACCAACCATTGAGCAGA-3’<br>R-5’-GATCATTGCTGAGGTGCTGTGTC-3’	101
JNK	F-5′AGTGTAGAGTGGATGCATGA3’<br>R-5′ATGTGCTTCCTGTGGTTTAC3’	182
β-actin	F-5′GGAGATTACTGCCCTGGCTCCTAGC3’<br>R-5′GGCCGGACTCATCGTACTCCTGCTT3’	155

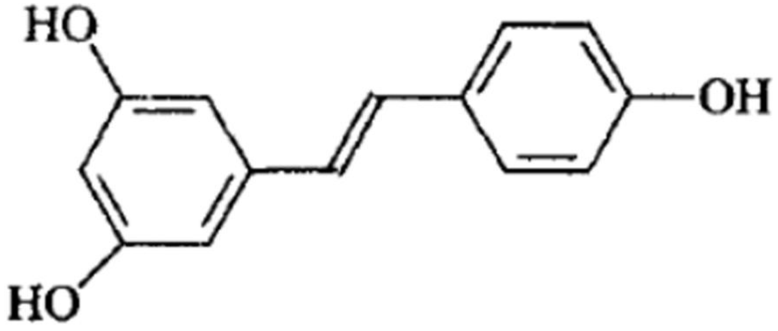

Supplementary Figure S1 Chemical structure of trans-RSV ($C_{14}H_{12}O_3$).

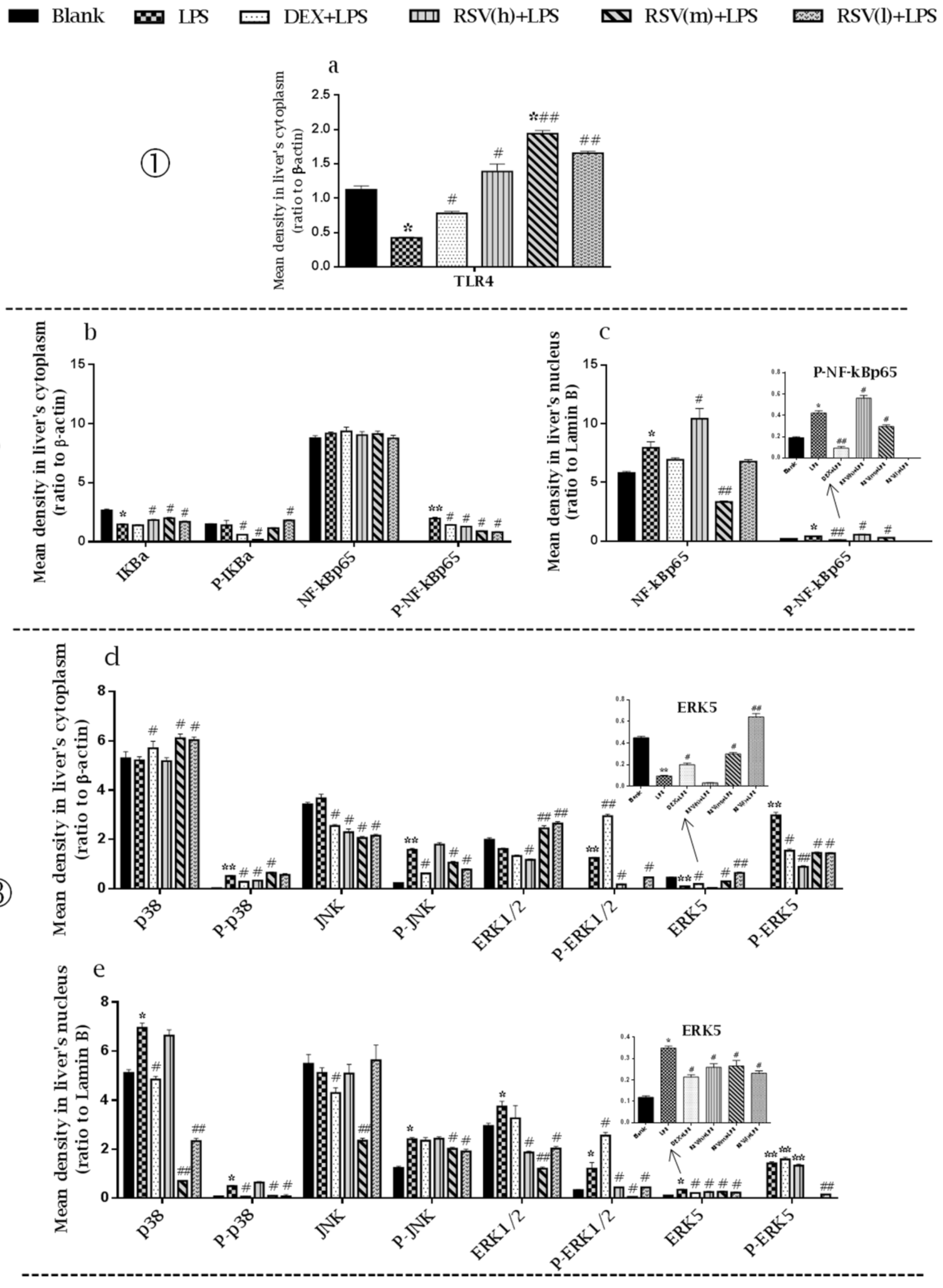
Blank
LPS
DEX+LPS
RSV(h)+LPS
RSV(m)+LPS
RSV(l)+LPS
a
①
Mean density in liver's cytoplasm (ratio to β-actin)
TLR4
②
b
Mean density in liver's cytoplasm (ratio to β-actin)
IKBa
P-IKBa
NF-kBp65
P-NF-kBp65
c
Mean density in liver's nucleus (ratio to Lamin B)
NF-kBp65
P-NF-kBp65
③
d
Mean density in liver's cytoplasm (ratio to β-actin)
ERK5
p38
P-p38
JNK
P-JNK
ERK1/2
P-ERK1/2
ERK5
P-ERK5
e
Mean density in liver's nucleus (ratio to Lamin B)
ERK5
p38
P-p38
JNK
P-JNK
ERK1/2
P-ERK1/2
ERK5
P-ERK5

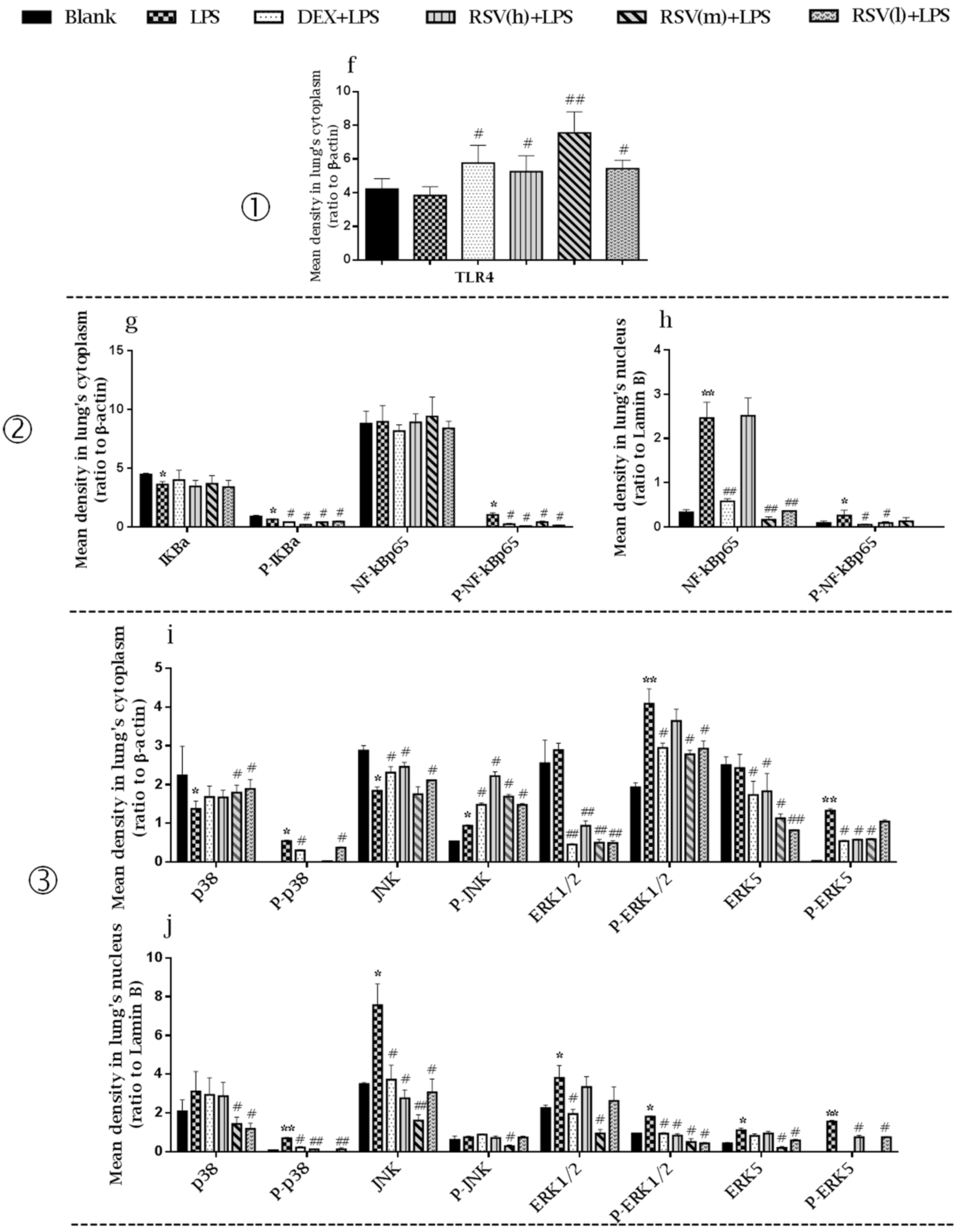

Supplementary Figure S2 Densitometric analysis of proteins and phosphorylated proteins in liver's and lung's cytoplasm and nucleus. ①: Mean density of related protein in TLR4 signaling pathway; ②: Mean density of related proteins and phosphorylated proteins in NF-κBp65 signaling pathway; ③: Mean density of related proteins and phosphorylated proteins in MAPKs signaling pathway. a to e : Mean density of proteins and phosphorylated proteins in liver's

cytoplasm and nucleus; f to j: Mean density of proteins and phosphorylated proteins in lung's cytoplasm and nucleus. DXM+LPS, RSV(h)+LPS, RSV(m)+LPS and RSV(l)+LPS represent the groups which pretreatment with DXM, high, intermediate and low dose of resveratrol before giving them LPS-stimulus, respectively. The values are presented as means ± standard deviation (10 rats /group). * $P<0.05$, ** $P<0.01$ vs. Blank control group; # $P<0.05$, ## $P<0.01$ vs. LPS group.
